# Microvessel Density as an Adjunctive Immunohistochemical Parameter in the Differential Diagnosis of Parathyroid Carcinoma and Adenoma—An Immunohistochemical Study

**DOI:** 10.3390/cancers18162650

**Published:** 2026-08-17

**Authors:** Zorka Inić, Katarina Taušanović, Marko Buta, Zoran Kozomara, Ognjen Živković, Nikola Jeftić, Stefan Gačić, Dobrica Stević, Anđela Milićević, Milan Žegarac

**Affiliations:** 1 Clinic for Surgical Oncology, Institute for Oncology and Radiology of Serbia, Pasterova 14, 11000 Belgrade, Serbiamilan_zegarac@yahoo.com (M.Ž.); 2 Faculty of Medicine, University of Belgrade, Dr Subotića 8, 11000 Belgrade, Serbia; 3 Clinic for Endocrine Surgery, University Clinical Center of Serbia, Pasterova 2, 11000 Belgrade, Serbia; 4 Department of Pathology, Institute for Oncology and Radiology of Serbia, Pasterova 14, 11000 Belgrade, Serbia

**Keywords:** parathyroid carcinoma, parathyroid adenoma, angiogenesis, microvessel density, immunohistochemistry, biomarkers, primary hyperparathyroidism, endocrine tumors

## Abstract

**Background/Objectives**: Distinguishing parathyroid carcinoma from parathyroid adenoma remains a major diagnostic challenge because these tumors frequently share similar clinical, biochemical, radiological, and histopathological features. Accurate diagnosis is essential for appropriate surgical management, highlighting the need for reliable adjunctive biomarkers. This study evaluated the diagnostic value of tumor angiogenesis, quantified by microvessel density (MVD), for differentiating parathyroid carcinoma from parathyroid adenoma and its association with clinicopathological characteristics. **Methods:** This retrospective study included 50 patients with primary hyperparathyroidism who underwent surgery at a tertiary endocrine surgery center, including 10 parathyroid carcinomas and 40 parathyroid adenomas. Tumor angiogenesis was assessed by immunohistochemical staining, and MVD was quantified in vascular hot spots using standardized methods. Receiver operating characteristic (ROC) analysis evaluated the diagnostic performance of MVD, while correlations with clinicopathological parameters were analyzed. **Results:** Parathyroid carcinoma demonstrated significantly higher MVD than parathyroid adenoma (median 901.14 vs. 431.24; *p* < 0.001). ROC analysis showed excellent diagnostic performance, with an area under the curve of 0.917. Higher MVD was positively associated with Ki-67 expression, preoperative parathyroid hormone and calcium levels, and tumor weight, indicating a close relationship between angiogenesis, tumor proliferation, biochemical disease severity, and tumor burden. No significant associations were observed with age or preoperative phosphate levels. **Conclusions:** Quantitative assessment of MVD is a promising adjunctive immunohistochemical biomarker for distinguishing parathyroid carcinoma from parathyroid adenoma. Incorporating MVD into conventional histopathological evaluation may improve diagnostic confidence, particularly in morphologically challenging cases. Larger multicenter studies are warranted to validate these findings and further establish the clinical utility of MVD.

## Simple Summary

Distinguishing parathyroid carcinoma (PC) from parathyroid adenoma (PA) remains a major diagnostic challenge because these tumors often share similar clinical, laboratory, and microscopic features. Accurate diagnosis is essential, as patients with parathyroid carcinoma usually require more extensive surgery and closer postoperative follow-up. In this study, we investigated whether tumor angiogenesis, assessed by measuring microvessel density, could help distinguish malignant from benign parathyroid tumors. We found that parathyroid carcinomas showed significantly higher microvessel density than adenomas, and increased vascularization was associated with higher cell proliferation, more severe biochemical abnormalities, and greater tumor burden. These findings suggest that quantitative assessment of microvessel density may complement conventional histopathological evaluation and improve diagnostic confidence in challenging cases. Further studies in larger patient populations are needed to validate its clinical usefulness.

## 1. Introduction

Parathyroid carcinoma (PC) is an exceptionally rare endocrine malignancy, accounting for less than 1% of all cases of primary hyperparathyroidism (PHPT) [1,2,3,4]. Despite its low incidence, it represents the most clinically aggressive parathyroid neoplasm because of its high rates of local recurrence, persistent hypercalcemia, and disease-related morbidity [1,2,3,5]. In contrast, parathyroid adenomas (PA), which account for approximately 80–85% of PHPT cases, is a benign lesion with an excellent prognosis following surgical excision [1,2,3]. Because complete en bloc resection at the initial operation offers the best chance for cure in PC, accurate differentiation between benign and malignant parathyroid tumors remains one of the greatest challenges in endocrine pathology and endocrine surgery [2,3,5,6].

The distinction between PA and PC is particularly difficult because these lesions frequently share overlapping clinical manifestations, biochemical abnormalities, radiological findings, and histopathological features [2,5,6,7]. Although unequivocal evidence of capsular, vascular, or perineural invasion, as well as distant metastasis, establishes the diagnosis of carcinoma, these criteria are often absent in early-stage disease [5,6,7,8]. Consequently, a substantial proportion of parathyroid carcinomas are initially classified as atypical adenomas or benign lesions, delaying appropriate treatment and increasing the risk of recurrence [2,6,9].

Tumor angiogenesis is a fundamental hallmark of cancer progression and plays a central role in tumor growth, invasion, and metastatic dissemination [10,11]. Quantitative assessment of angiogenesis by measuring microvessel density (MVD) has emerged as a reproducible indicator of tumor vascularization and has demonstrated diagnostic and prognostic value in several malignancies, including breast, colorectal, thyroid, pancreatic, and neuroendocrine tumors [11,12,13].

In parathyroid neoplasms, however, the biological and diagnostic significance of angiogenesis remains incompletely understood [14,15,16]. Previous studies evaluating vascular endothelial growth factor (VEGF), CD31, CD34, and other endothelial markers have yielded inconsistent findings, largely because of the rarity of parathyroid carcinoma and the limited size of available cohorts [14,15,16]. As a result, reliable biomarkers capable of improving the differential diagnosis between parathyroid adenoma and carcinoma are still lacking [17,18,19,20,21,22].

Recent advances in endocrine pathology have emphasized the value of integrating conventional histopathology with immunohistochemical and molecular biomarkers [17,18,19,20,21]. Several markers, including parafibromin, Ki-67, galectin-3, PGP9.5, APC, and other molecular alterations, have improved diagnostic precision; however, none has demonstrated sufficient sensitivity and specificity to be used as a standalone diagnostic test [17,18,19,20,21]. Current evidence therefore supports a multiparametric approach that combines histomorphological assessment with objective immunohistochemical evaluation [5,18,19,20,21].

Microvessel density is one of the most widely accepted methods for quantifying tumor angiogenesis and provides objective information on tumor vascularization and biological behavior [11,12,13].

The present study evaluated tumor angiogenesis in surgically treated parathyroid neoplasms by investigating whether quantitative assessment of CD105-defined microvessel density (MVD) may provide supportive information in the differential diagnosis of parathyroid carcinoma and parathyroid adenoma. We hypothesized that malignant parathyroid tumors would exhibit greater angiogenic activity than benign lesions. Therefore, the aim of the present study was to assess CD105-defined MVD in parathyroid carcinoma and adenoma and to explore its potential value as an adjunctive immunohistochemical parameter in their differential diagnosis. We hypothesized that malignant parathyroid tumors exhibit significantly greater angiogenesis than benign lesions. Therefore, the aim of the present study was to determine whether quantitative assessment of CD105-defined microvessel density can improve the differential diagnosis between parathyroid carcinoma and parathyroid adenoma.

## 2. Materials and Methods

### 2.1. Study Design

This single-center retrospective observational study was conducted to investigate the diagnostic significance of tumor angiogenesis in parathyroid neoplasms. The study included consecutive patients who underwent surgery for primary hyperparathyroidism at the Center for Endocrine Surgery, University Clinical Center of Serbia, Belgrade, Serbia over a 10-year period. Archived formalin-fixed, paraffin-embedded (FFPE) tissue specimens were retrieved from the Department of Pathology for histopathological and immunohistochemical analyses. FFPE blocks were stored under routine archival conditions in the institutional pathology archive. Given the archival nature of the material, preservation of CD105 immunoreactivity was assessed using internal endothelial structures as positive controls for each tissue specimen, in addition to the external positive and negative controls included in each immunohistochemical staining run.

The primary objective was to determine whether quantitative assessment of MVD could provide supportive information in the differential diagnosis of parathyroid carcinoma and parathyroid adenoma.

The study was conducted in accordance with the Declaration of Helsinki [23].

### 2.2. Study Population

A total of 60 parathyroid tissue specimens were analyzed. According to the final histopathological diagnosis, patients were allocated into three groups:

- parathyroid carcinoma (PC; n = 10);
- parathyroid adenoma (PA; n = 40);
- normal parathyroid tissue (control group; n = 10).

Only patients with complete clinical documentation, biochemically confirmed primary hyperparathyroidism, complete surgical excision of the lesion, histopathological confirmation of parathyroid adenoma or carcinoma, available FFPE tissue blocks, and adequate material for immunohistochemical analysis were included.

Exclusion criteria comprised secondary or tertiary hyperparathyroidism, hereditary forms of primary hyperparathyroidism (including multiple endocrine neoplasia [MEN] syndromes and familial isolated hyperparathyroidism), recurrent disease without available primary tumor tissue, insufficient tissue for immunohistochemical analysis, and incomplete clinical or medical records.

Normal parathyroid glands were obtained from patients undergoing thyroidectomy or hemithyroidectomy in whom macroscopically and microscopically normal parathyroid tissue was incidentally removed.

Clinical, biochemical, and imaging data were retrieved from the institutional medical records, including patient age, sex, lesion localization, preoperative serum parathyroid hormone (PTH), total calcium, inorganic phosphate concentrations, and scintigraphic findings.

### 2.3. Histopathological Evaluation

All surgical specimens were fixed in 10% neutral-buffered formalin, routinely processed, embedded in paraffin, and sectioned at 4-μm thickness. Hematoxylin and eosin (H&E)-stained sections were reviewed independently by two experienced pathologists blinded to the immunohistochemical findings.

The diagnosis of parathyroid carcinoma was established according to internationally accepted histopathological criteria, including capsular invasion, vascular invasion, perineural invasion, infiltration of adjacent soft tissues, local recurrence, or metastatic disease [5,8]. Cases lacking unequivocal evidence of malignancy were classified as parathyroid adenomas according to established diagnostic criteria.

### 2.4. Immunohistochemistry

Immunohistochemical staining was performed on FFPE tissue sections using the avidin–biotin complex (ABC) method (Vectastain ABC Kit, Vector Laboratories, Burlingame, CA, USA) [17,21].

Following deparaffinization and rehydration, antigen retrieval was performed by heat-induced epitope retrieval in citrate buffer (pH 6.0). Endogenous peroxidase activity was blocked using 3% hydrogen peroxide. Sections were subsequently incubated with primary anti-CD105 (endoglin) and anti-Ki-67 antibodies to evaluate tumor angiogenesis and proliferative activity, respectively, according to the manufacturer’s instructions. Immunoreactivity was visualized using 3,3′-diaminobenzidine (DAB) as the chromogen and counterstained with Mayer’s hematoxylin.

Positive and negative control tissues were included in each staining run to ensure staining quality and reproducibility. In addition, endothelial cells lining blood vessels present within the tissue sections served as internal positive controls for CD105 immunoreactivity, confirming adequate staining in each specimen.

### 2.5. Assessment of Microvessel Density

Tumor angiogenesis was evaluated by quantitative assessment of MVD using the method originally described by Weidner et al. [9,10]. The entire tumor section was initially examined at low magnification (×40–×100) to identify areas with the highest vascular density (“hot spots”). Microvessels were subsequently counted in three representative hot spots at ×200 magnification.

Any brown-stained endothelial cell or endothelial cell cluster clearly separated from adjacent vessels and connective tissue elements was considered an individual microvessel, irrespective of the presence of a visible vascular lumen. Large muscular vessels and vessels located outside the tumor tissue were excluded from the analysis.

The same objective magnification and predefined field-selection and vessel-counting criteria were applied to all specimens. The mean number of microvessels counted in the three selected hot spots was recorded as the MVD value for each case. All evaluations were independently performed by two pathologists blinded to the final diagnosis. Any discrepancies were resolved by joint review and consensus.

Because the original archival protocol did not document the corresponding microscopic field area in mm^2^, MVD values are reported according to the original standardized field-based counting method.

### 2.6. Statistical Analysis

Statistical analyses were performed using IBM SPSS Statistics version 15.0 (IBM Corp., Armonk, NY, USA).

Continuous variables were expressed as mean ± standard deviation (SD) or median with interquartile range (IQR), according to data distribution, while categorical variables were presented as absolute numbers and percentages.

Normality of data distribution was assessed using the Shapiro–Wilk and Kolmogorov–Smirnov tests. Differences between groups were analyzed using Student’s *t*-test or the Mann–Whitney U test for continuous variables and the χ^2^ test or Fisher’s exact test for categorical variables. Correlations between continuous variables were evaluated using linear correlation analysis, and multivariable linear regression analysis was performed where appropriate.

Receiver operating characteristic (ROC) curve analysis was used to assess the discriminatory performance of MVD in differentiating parathyroid carcinoma from parathyroid adenoma. The area under the curve (AUC), sensitivity, specificity, positive predictive value, negative predictive value, and study-specific ROC-derived cutoff value determined by the Youden index were calculated.

A two-sided *p*-value < 0.05 was considered statistically significant.

## 3. Results

### 3.1. Patient Demographics

A total of 50 consecutive patients with primary hyperparathyroidism who underwent surgical treatment at a tertiary referral endocrine surgery center were included in the study, including 10 patients with parathyroid carcinoma and 40 patients with parathyroid adenoma. An additional 10 histologically normal parathyroid glands served as the control group for immunohistochemical analyses.

Baseline demographic and clinicopathological characteristics of the study population are presented in Table 1. No significant differences in age distribution were observed between patients with parathyroid carcinoma and those with parathyroid adenoma (ANOVA, F = 1.841, *p* = 0.168).

### 3.2. Clinicopathological Characteristics

#### 3.2.1. Preoperative Scintigraphic Findings

Preoperative parathyroid scintigraphy most frequently demonstrated increased radiotracer uptake within the affected gland, which was observed in 62.0% of all patients. This imaging pattern was present in 60.0% of carcinomas and 62.5% of adenomas.

Hyperfunctional lesions were identified exclusively in patients with adenoma (37.5%), whereas non-functioning lesions were observed only in the carcinoma group (40.0%). Overall, scintigraphic findings showed substantial overlap between the two pathological entities and therefore had limited value for distinguishing parathyroid carcinoma from adenoma.

#### 3.2.2. Tumor Size and Weight

Parathyroid carcinomas were significantly larger than adenomas. The mean tumor diameter was 37.9 ± 7.7 mm in carcinomas compared with 20.9 ± 7.6 mm in adenomas, representing an almost two-fold difference. Student’s *t*-test confirmed that this difference was highly statistically significant (*t* = 6.291, *p* < 0.001).

Similarly, tumor weight differed markedly between the two groups. Carcinomas had a mean weight of 11.77 ± 8.92 g (median, 8.1 g; range, 3.5–28.0 g), whereas adenomas had a mean weight of 1.69 ± 2.13 g (median, 0.9 g; range, 0.2–10.2 g). Mann–Whitney analysis demonstrated a highly significant difference in tumor weight between carcinomas and adenomas (U = 16.00, Z = 4.472, *p* < 0.001).

Both tumor diameter and tumor weight were substantially greater in parathyroid carcinoma, indicating that macroscopic tumor characteristics may provide valuable adjunctive information when differentiating malignant from benign parathyroid neoplasms.

### 3.3. Histopathological Findings

Histopathological examination confirmed the diagnosis of parathyroid adenoma and parathyroid carcinoma in all cases. The diagnosis of parathyroid carcinoma was based on established histopathological criteria, with particular emphasis on unequivocal vascular invasion, perineural invasion, invasion of adjacent structures, and/or metastatic disease, as applicable. Additional histopathological features, including prominent fibrous bands and a trabecular growth pattern, were also assessed as supportive morphological features. In contrast, parathyroid adenomas demonstrated typical benign histomorphological features without evidence of capsular or vascular invasion.

### 3.4. Immunohistochemical Findings

#### 3.4.1. Ki-67 Expression

**Figure 1:**
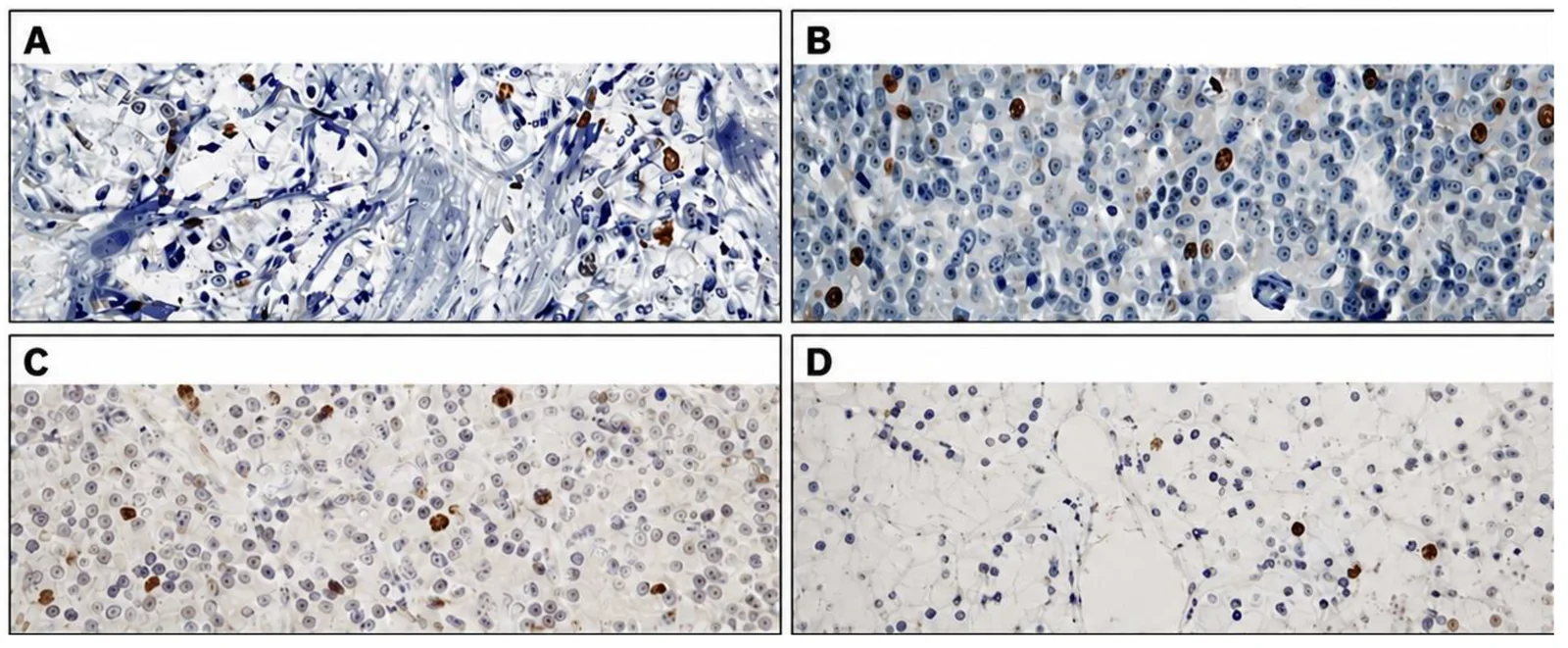
Ki-67 immunohistochemical expression in parathyroid carcinoma and adenoma. (**A**) Ki-67 immunostaining in parathyroid carcinoma at ×200 magnification. (**B**) Higher-power view of Ki-67 immunostaining in parathyroid carcinoma at ×400 magnification. (**C**) Ki-67 immunostaining in parathyroid adenoma at ×200 magnification. (**D**) Higher-power view of Ki-67 immunostaining in parathyroid adenoma at ×400 magnification.

**Figure 2:**
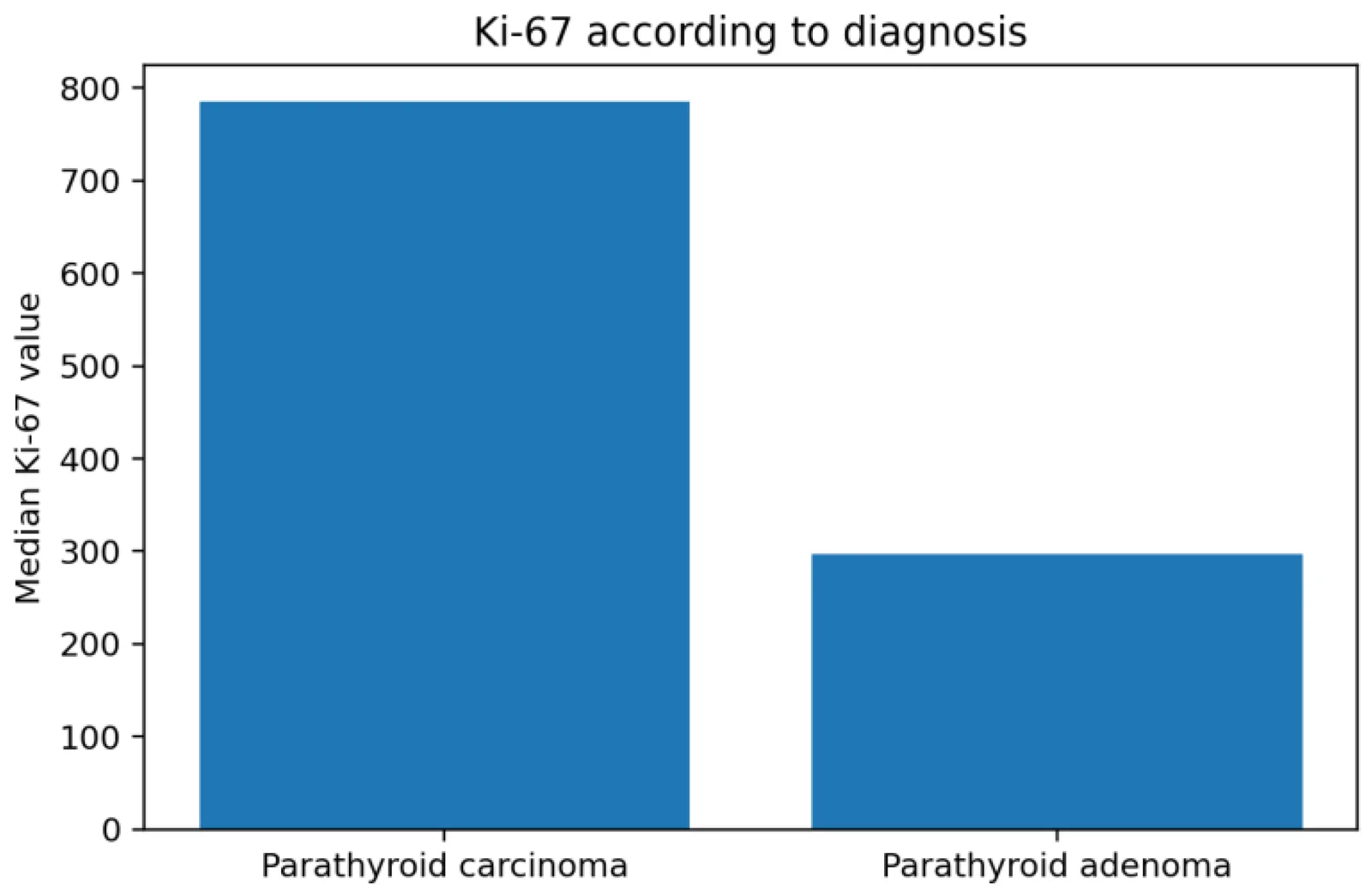
Median Ki-67 values according to diagnosis.

Quantitative analysis demonstrated significantly higher Ki-67 expression in parathyroid carcinoma than in parathyroid adenoma (Figure 1). The mean Ki-67 value was 1024.27 ± 564.19 in carcinomas compared with 334.66 ± 161.71 in adenomas, while the median values were 785.15 and 297.41, respectively (Figure 2). Mann–Whitney analysis confirmed a highly significant difference between the two groups (U = 10.50, Z = 4.598, *p* < 0.001) (Table 2).

These findings indicate substantially increased proliferative activity in parathyroid carcinoma compared with adenoma, supporting the potential value of Ki-67 as an adjunctive immunohistochemical parameter for distinguishing malignant from benign parathyroid tumors.

#### 3.4.2. Microvessel Density

**Figure 3:**
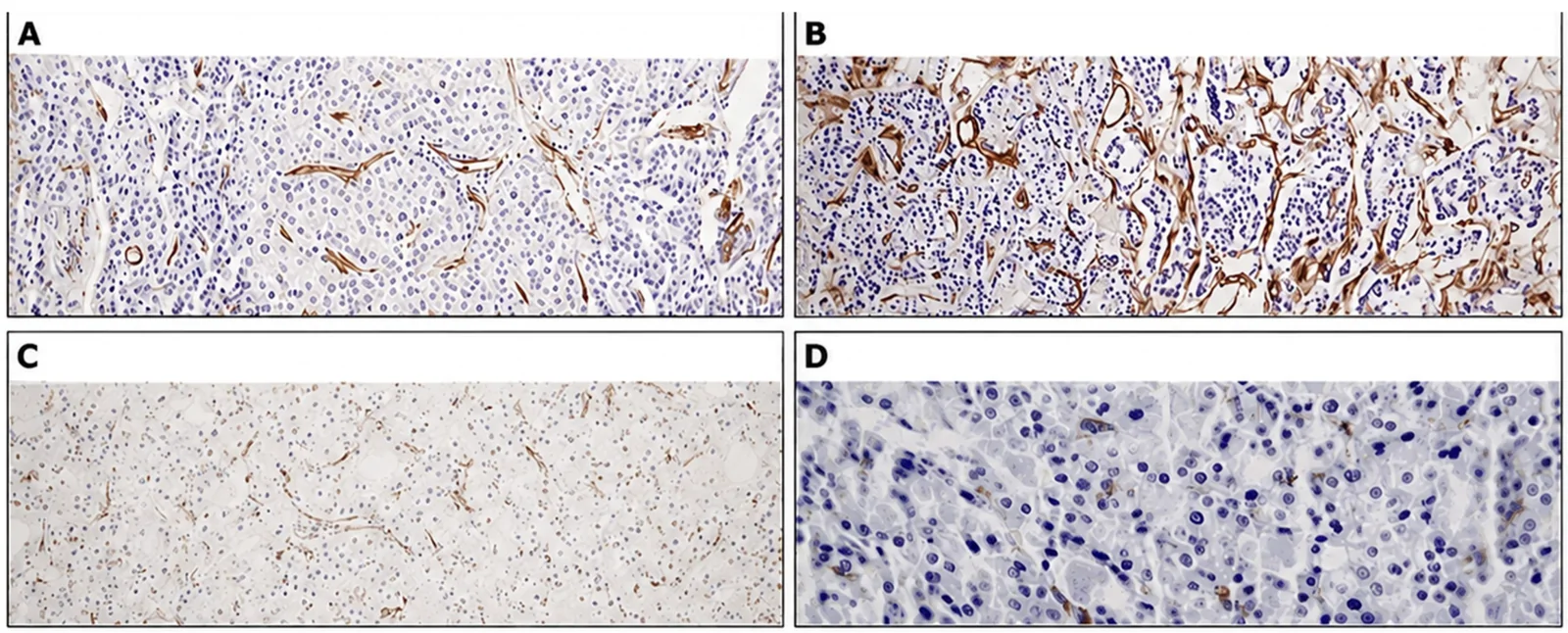
CD105 immunohistochemical staining in parathyroid carcinoma and adenoma. (**A**) Parathyroid carcinoma showing CD105-positive microvessels at ×200 magnification. (**B**) Higher-power view of CD105-positive microvessels in parathyroid carcinoma at ×400 magnification. (**C**) Parathyroid adenoma showing CD105-positive microvessels at ×200 magnification. (**D**) Higher-power view of CD105-positive microvessels in parathyroid adenoma at ×400 magnification.

**Figure 4:**
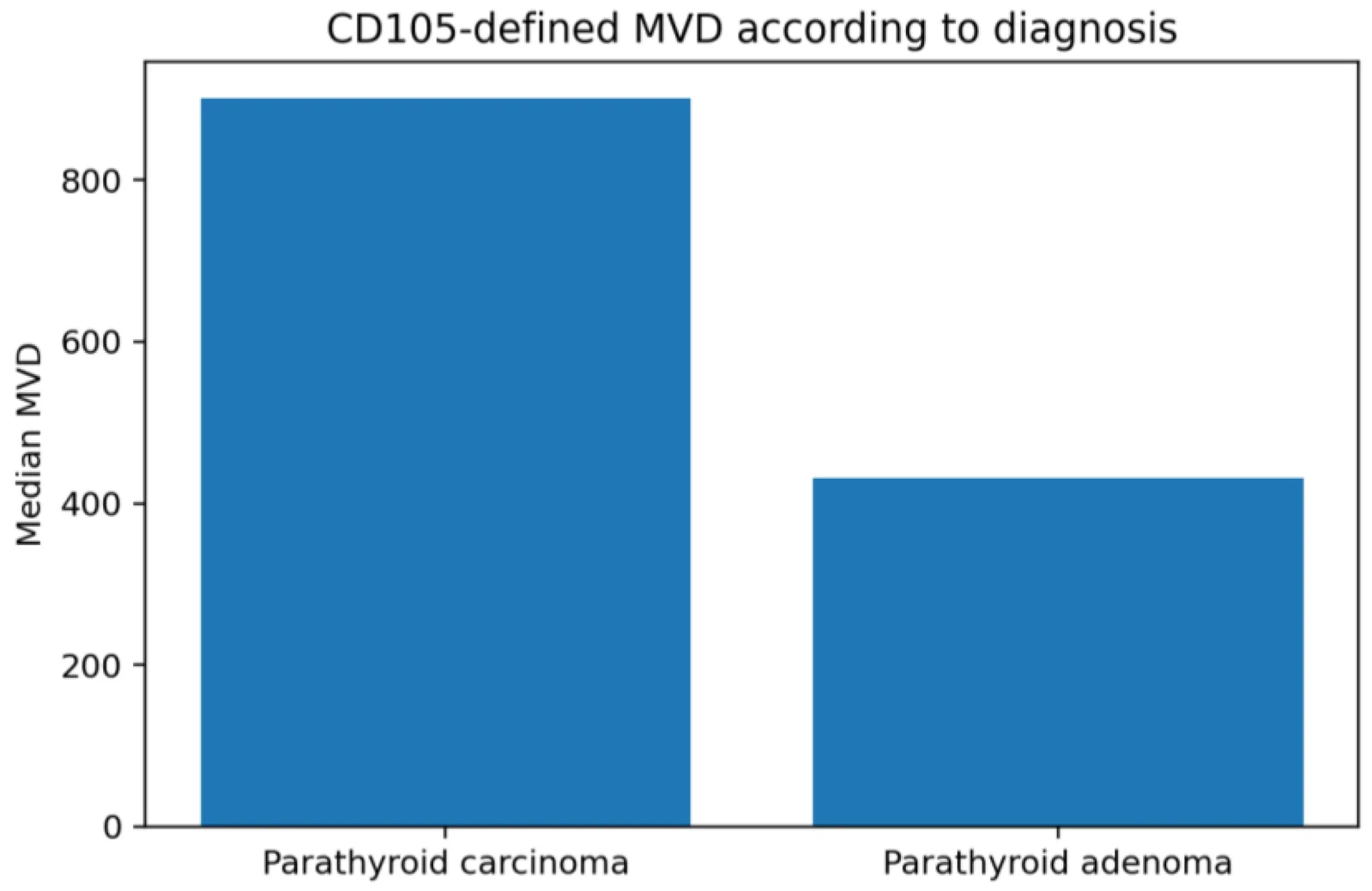
Median CD105-defined microvessel density according to diagnosis.

MVD, assessed by CD105 immunohistochemistry, was also significantly higher in parathyroid carcinoma than in adenoma (Figure 3). Carcinomas demonstrated a mean MVD of 830.36 ± 224.06 and a median value of 901.14, whereas adenomas showed a mean value of 447.22 ± 211.63 and a median value of 431.24 (Figure 4). The difference between the two groups was highly statistically significant (Mann–Whitney U = 41.50, Z = 3.845, *p* < 0.001). The increased MVD observed in carcinomas indicates significantly enhanced tumor angiogenesis compared with benign parathyroid neoplasms (Table 3).

The normal parathyroid control specimens from the original investigation were not included in the carcinoma-versus-adenoma diagnostic comparison.

#### 3.4.3. Receiver Operating Characteristic (ROC) Analysis

ROC curve analysis was performed to evaluate the discriminatory performance of Ki-67 expression and MVD in differentiating parathyroid carcinoma from parathyroid adenoma.

Ki-67 demonstrated excellent discriminatory performance, with an area under the ROC curve AUC of 0.979 (95% CI, 0.947–1.000; *p* < 0.001). The study-specific ROC-derived cutoff for Ki-67 was >511.54, yielding a sensitivity of 100.0% and a specificity of 86.0%. In this cohort, Ki-67 values above this study-specific threshold were associated with parathyroid carcinoma.

MVD also showed excellent discriminatory ability, with an AUC of 0.917 (95% CI, 0.809–1.000; *p* < 0.001). Using a study-specific ROC-derived cutoff of >773.26, MVD achieved a sensitivity of 80.0% and a specificity of 100.0%.

Comparison of the ROC curves demonstrated that both Ki-67 and MVD demonstrated strong discriminatory performance for differentiating parathyroid carcinoma from adenoma in this cohort, although these findings require validation in larger independent cohorts. However, Ki-67 exhibited superior overall performance, reflected by a larger AUC and higher sensitivity, whereas MVD provided maximal specificity. These findings suggest that assessment of both proliferative activity and tumor angiogenesis may improve the histopathological differentiation of malignant and benign parathyroid tumors.

#### 3.4.4. Ki-67 and Microvessel Density

Ki-67-positive cell density was significantly higher in parathyroid carcinoma than in adenoma (median, 785.15 vs. 297.41, Mann–Whitney U = 10.5, Z = 4.598, *p* < 0.001). Similarly, MVD was significantly increased in carcinoma compared with adenoma (median, 901.14 vs. 431.24, Mann–Whitney U = 41.5, Z = 3.845, *p* < 0.001).

Receiver operating characteristic (ROC) analysis demonstrated strong discriminatory performance of both parameters for differentiating parathyroid carcinoma from adenoma. The area under the ROC curve (AUC) for Ki-67-positive cell density was 0.979 (95% CI, 0.947–1.000; *p* < 0.001), whereas the AUC for MVD was 0.917 (95% CI, 0.809–1.000; *p* < 0.001).

The study-specific ROC-derived cutoff for Ki-67-positive cell density was >511.54, yielding a sensitivity of 100% and a specificity of 86%. For MVD, the study-specific ROC-derived cutoff was >773.26, providing a sensitivity of 80% and a specificity of 100%. These findings indicate that both Ki-67-positive cell density and MVD demonstrated strong discriminatory performance for differentiating parathyroid carcinoma from adenoma in this cohort, with Ki-67-positive cell density showing a higher AUC than MVD.

### 3.5. Biochemical Findings

#### 3.5.1. Preoperative Parathyroid Hormone Levels

Preoperative serum PTH levels were significantly higher in patients with parathyroid carcinoma than in those with parathyroid adenoma. The carcinoma group demonstrated a mean preoperative PTH concentration of 1824.02 ± 861.47 pg/mL (median, 1721.0 pg/mL), whereas patients with adenoma had a mean concentration of 266.04 ± 235.75 pg/mL (median, 189.5 pg/mL). Because PTH values were not normally distributed, comparisons between groups were performed using the Mann–Whitney U test, which revealed a highly significant difference (U = 9.00, Z = 4.361, *p* < 0.001).

Postoperative PTH levels remained significantly higher in patients with carcinoma than in those with adenoma (median, 78.2 vs. 19.5 pg/mL; U = 51.00, Z = 3.614, *p* < 0.001). However, both groups exhibited a marked postoperative reduction in circulating PTH concentrations. Wilcoxon signed-rank analysis confirmed a significant decline in PTH following surgery in patients with carcinoma (Z = 2.803, *p* = 0.005) as well as in those with adenoma (Z = 5.551, *p* < 0.001), demonstrating the effectiveness of surgical treatment in both groups.

#### 3.5.2. Serum Calcium Levels

Preoperative serum calcium concentrations were significantly elevated in patients with parathyroid carcinoma compared with those with parathyroid adenoma (3.55 ± 0.59 vs. 2.88 ± 0.21 mmol/L, *p* < 0.001). Following surgery, serum calcium levels decreased significantly in both groups, reaching mean postoperative values of 2.31 ± 0.37 mmol/L in the carcinoma group and 2.33 ± 0.20 mmol/L in the adenoma group.

Repeated-measures analysis of variance demonstrated significant effects of time (F = 148.906, *p* < 0.001), diagnosis (F = 22.800, *p* < 0.001), and the interaction between these two factors (F = 23.033, *p* < 0.001). Pairwise comparisons using Sidak’s correction confirmed significantly higher preoperative calcium levels in carcinoma than in adenoma (*p* < 0.001), whereas no significant difference was observed after surgery (*p* = 0.754). These findings indicate that surgical treatment successfully normalized serum calcium concentrations regardless of tumor type.

#### 3.5.3. Serum Phosphate Levels

Serum phosphate concentrations increased significantly following surgery in both patient groups. In the carcinoma group, mean phosphate levels increased from 0.73 ± 0.34 mmol/L preoperatively to 1.04 ± 0.34 mmol/L postoperatively, while corresponding values in the adenoma group increased from 0.80 ± 0.18 mmol/L to 1.06 ± 0.24 mmol/L.

Repeated-measures ANOVA demonstrated a significant effect of time (F = 50.965, *p* < 0.001), indicating postoperative normalization of phosphate metabolism. In contrast, neither the effect of diagnosis (F = 0.310, *p* = 0.580) nor the interaction between diagnosis and time (F = 0.310, *p* = 0.531) reached statistical significance, suggesting comparable phosphate dynamics in patients with carcinoma and adenoma.

#### 3.5.4. Receiver Operating Characteristic Analysis of Biochemical Parameters

ROC curve analysis was performed to evaluate the diagnostic performance of preoperative biochemical parameters for distinguishing parathyroid carcinoma from parathyroid adenoma.

Preoperative serum PTH demonstrated excellent diagnostic accuracy, with an AUC of 0.978 (95% CI, 0.939–1.000; *p* < 0.001). The optimal diagnostic threshold was >768 pg/mL, corresponding to a sensitivity of 90.0% and a specificity of 97.5%, indicating that markedly elevated preoperative PTH levels strongly predict parathyroid carcinoma.

Preoperative serum calcium also showed good diagnostic performance, with an AUC of 0.864 (95% CI, 0.733–0.995; *p* < 0.001). A cut-off value of >2.555 mmol/L yielded a sensitivity of 90.0% and a specificity of 75.0%.

Overall, preoperative PTH outperformed serum calcium in discriminating parathyroid carcinoma from adenoma, as reflected by its higher AUC and specificity. These findings support the combined use of biochemical markers together with histopathological and immunohistochemical parameters in the preoperative assessment of patients with primary hyperparathyroidism (Table 4).

**Table 4:** ROC characteristics of Ki-67, MVD, preoperative PTH, and preoperative.

Marker	AUC	SE	*p* Value	95% CI	Cut off	Sensitivity	Specificity
Ki-67	0.979	0.016	<0.001	0.947–1.000	>511.54	100%	86%
MVD	0.917	0.055	<0.001	0.809–1.000	>773.26	80%	100%
Preoperative PTH	0.978	0.020	<0.001	0.939–1.000	>768	90%	97.5%
Preoperative calcium	0.864	0.067	<0.001	0.733–0.995	>2.555 mmol/L	90%	75%

### 3.6. Correlation and Regression Analyses

**Figure 5:**
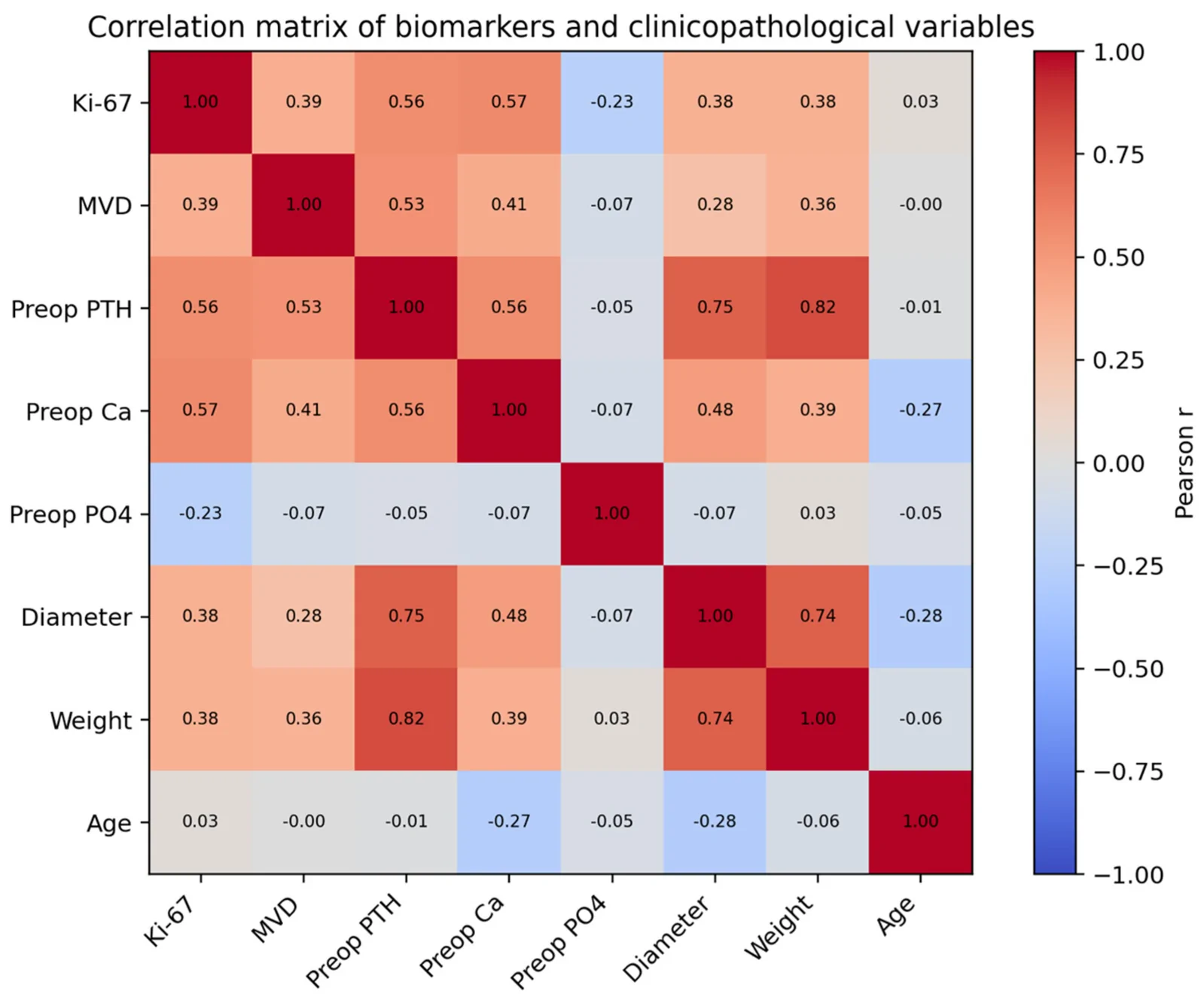
Correlation matrix of immunohistochemical and clinicopathological variables.

Pearson correlation analysis demonstrated significant positive associations between Ki-67 expression and MVD (r = 0.389, *p* = 0.005), preoperative PTH (r = 0.562, *p* < 0.001), preoperative calcium (r = 0.571, *p* < 0.001), tumor diameter (r = 0.378, *p* = 0.007), and tumor weight (r = 0.382, *p* = 0.006). No significant correlations were observed between Ki-67 and patient age or preoperative phosphate levels (Table 5, Figure 5).

MVD was positively associated with preoperative PTH (r = 0.534, *p* < 0.001), preoperative calcium (r = 0.406, *p* = 0.003), and tumor weight (r = 0.356, *p* = 0.011). Preoperative PTH exhibited the strongest correlations with tumor weight (r = 0.820, *p* < 0.001) and tumor diameter (r = 0.748, *p* < 0.001), while preoperative calcium correlated significantly with both tumor diameter (r = 0.477, *p* < 0.001) and tumor weight (r = 0.388, *p* = 0.005). Neither patient age nor preoperative phosphate levels showed significant correlations with any evaluated parameter.

Multiple linear regression analysis was performed using Ki-67 as the dependent variable and preoperative PTH, preoperative calcium, tumor diameter, and tumor weight as independent variables. The model explained 42.3% of the variability in Ki-67 expression (R^2^ = 0.423; adjusted R^2^ = 0.372) and was statistically significant (F = 8.249, *p* < 0.001). Preoperative PTH (B = 0.274, *p* = 0.027) and preoperative calcium (B = 351.566, *p* = 0.012) emerged as independent predictors of Ki-67 expression, whereas tumor diameter and tumor weight were not independently associated with Ki-67 after adjustment.

All patients demonstrated elevated preoperative PTH concentrations. Elevated serum calcium was present in all patients with parathyroid carcinoma and in 90% of those with adenoma, whereas elevated phosphate levels were observed in 70% and 47.5% of patients, respectively. Neither difference reached statistical significance (Fisher’s exact test, *p* = 0.397 and *p* = 0.179, respectively).

## 4. Discussion

The principal finding of the present study is that parathyroid carcinoma is characterized by significantly increased CD105-assessed MVD compared with parathyroid adenoma, indicating substantially enhanced tumor angiogenesis in malignant parathyroid neoplasms. Quantitative assessment of MVD demonstrated excellent diagnostic performance (AUC = 0.917), highlighting tumor angiogenesis as a promising adjunctive biomarker for differentiating malignant from benign parathyroid tumors. Beyond its diagnostic value, increased MVD was positively associated with Ki-67 proliferative activity, preoperative serum PTH and calcium concentrations, and tumor weight, suggesting that angiogenesis is closely linked to tumor proliferation, endocrine activity, and tumor burden. These findings are consistent with the established role of angiogenesis as a hallmark of cancer progression and a key determinant of tumor growth, invasion, and biological aggressiveness [10,11,12,13]. Previous studies investigating angiogenesis in parathyroid neoplasms have likewise suggested increased vascularization in parathyroid carcinoma, although the available evidence remains limited because of the rarity of this malignancy and the small size of published cohorts [14,15,16]. Collectively, our findings support the concept that neovascularization represents an integral component of the aggressive biological phenotype of parathyroid carcinoma and further reinforce the value of combining quantitative immunohistochemical biomarkers with conventional histopathological assessment to improve diagnostic accuracy [5,17,18,19,20,21,22].

### 4.1. Biological Significance of Angiogenesis

The biological significance of increased microvessel density in parathyroid carcinoma is supported by the unique characteristics of CD105 (endoglin), a transmembrane glycoprotein that is preferentially expressed on proliferating endothelial cells involved in active neoangiogenesis [24]. Unlike pan-endothelial markers such as CD31 and CD34, which stain both mature and newly formed vessels, CD105 is preferentially expressed on activated proliferating endothelial cells associated with tumor neoangiogenesis, thereby providing a more specific assessment of ongoing angiogenic activity [25]. This distinction is particularly important in parathyroid neoplasms, where reliable assessment of tumor-associated angiogenesis remains challenging because of the rarity of parathyroid carcinoma and the limited number of published studies [14,15]. The significantly increased CD105-defined MVD observed in our cohort therefore suggests that malignant parathyroid tumors undergo active vascular remodeling, reflecting the increased metabolic demands associated with rapid cellular proliferation, sustained hormone production, and invasive tumor growth. This observation is biologically plausible because tumor angiogenesis is orchestrated by a complex network of pro-angiogenic signaling pathways that promote endothelial cell proliferation, vascular remodeling, and sustained neovascularization, thereby facilitating tumor growth and progression [11]. Accordingly, the increased CD105 expression observed in parathyroid carcinoma probably reflects not only the increased metabolic requirements of malignant tumors but also a more aggressive biological phenotype characterized by enhanced angiogenic activity [11]. This interpretation is further supported by previous studies reporting increased vascularization in parathyroid neoplasms, despite considerable methodological heterogeneity and the limited number of investigated cases [14,15]. Taken together, these findings support the concept that enhanced angiogenesis is not merely a secondary feature of malignant parathyroid tumors but an integral component of their aggressive biological behavior.

### 4.2. Comparison with Previous Studies

Our findings are broadly consistent with the limited body of literature investigating angiogenesis in parathyroid neoplasms. Garcia de la Torre et al. demonstrated increased angiogenic and lymphangiogenic activity in proliferative parathyroid lesions, suggesting that vascular remodeling accompanies parathyroid tumorigenesis [15]. Similarly, Viacava et al. reported differences in microvessel density between normal and neoplastic parathyroid tissues, whereas Segiet et al. further emphasized the potential contribution of angiogenesis to the pathogenesis of primary hyperparathyroidism [14,16]. Nevertheless, the available evidence remains heterogeneous, and not all studies have demonstrated statistically significant differences in microvessel density between parathyroid carcinoma and adenoma. These discrepancies most likely reflect methodological differences, including variation in endothelial markers, immunohistochemical protocols, hotspot selection, methods of vessel quantification, and the inherently small number of parathyroid carcinoma cases available for analysis. Additional sources of variability include differences in patient selection, diagnostic criteria, and the exceptionally low incidence of parathyroid carcinoma, which has limited the size and statistical power of virtually all published series [14,15,16]. In this context, our findings further support active angiogenesis as a characteristic feature of parathyroid carcinoma and suggest that CD105 may provide greater biological specificity than conventional pan-endothelial markers for evaluating tumor-associated neovascularization [25]. However, direct comparisons between parathyroid carcinoma and adenoma remain limited because of the exceptional rarity of parathyroid carcinoma and the consequently small size of published cohorts [2,4,26]. Taken together, the available evidence and the findings of the present study support CD105-defined MVD as a reproducible and biologically meaningful measure of tumor angiogenesis, although it should be regarded as an adjunctive rather than standalone diagnostic marker [5,6,17,18,19,20,21,22].

### 4.3. Diagnostic Implications of CD105-Defined Microvessel Density

Beyond its biological significance, the present study demonstrated that CD105-defined microvessel density demonstrated strong discriminatory performance in differentiating parathyroid carcinoma from parathyroid adenoma in the present cohort. ROC analysis yielded an AUC of 0.917, with a study-specific ROC-derived cutoff of >773.26 providing 80% sensitivity and 100% specificity. However, this threshold should not be considered a clinically validated cutoff because of the small number of parathyroid carcinoma cases and the retrospective single-center design of the study. Although Ki-67 demonstrated a slightly higher AUC (0.979), the high specificity observed for CD105-defined MVD suggests that increased tumor angiogenesis may provide useful supportive information in the differential diagnosis of parathyroid carcinoma and adenoma. Rather than serving as a standalone diagnostic marker, CD105-defined MVD should be interpreted as an adjunctive immunohistochemical parameter in conjunction with conventional histopathological findings and established diagnostic markers [5,17,18,19,20,21,22]. This multiparametric approach is particularly relevant in morphologically challenging lesions, including atypical parathyroid tumors and carcinomas lacking unequivocal invasive features, in which reliance on a single biomarker may be insufficient. Accordingly, quantitative assessment of tumor angiogenesis may contribute to a more objective and reproducible diagnostic algorithm for parathyroid neoplasms. The excellent specificity observed in the present study suggests that markedly increased CD105-defined MVD may provide supportive information in the differential diagnosis of parathyroid neoplasms, particularly when interpreted together with conventional histopathological findings and established immunohistochemical markers [5,17,18,19,20,21,22]. The moderate sensitivity observed in the present study further supports interpreting MVD as an adjunctive parameter within a multiparametric diagnostic approach rather than as a standalone test.

### 4.4. Interrelationship Between Angiogenesis, Proliferative Activity, Biochemical Severity, and Tumor Burden

In addition to distinguishing parathyroid carcinoma from parathyroid adenoma, the present study demonstrated that angiogenesis, cellular proliferation, biochemical disease activity, and tumor burden represent closely interconnected components of the malignant phenotype [10,11,12,13]. Rather than occurring as isolated pathological events, these biological processes appear to evolve in parallel during tumor progression, reflecting coordinated activation of mechanisms that support endocrine hyperfunction, sustained cellular proliferation, and neovascularization.

Among the evaluated clinicopathological variables, preoperative serum PTH demonstrated the strongest associations with tumor diameter and tumor weight, indicating that hormonally active tumors are also those with the greatest tumor burden. These findings are consistent with previous clinical studies showing that parathyroid carcinoma is typically characterized by markedly elevated serum PTH concentrations, severe hypercalcemia, and substantially larger tumors than benign adenomas [1,2,4,9,27]. Collectively, these observations support the concept that increasing biochemical severity parallels tumor progression and reflects the aggressive biological behavior of parathyroid carcinoma.

The significant positive correlations observed between CD105-defined microvessel density, Ki-67 expression, serum PTH, calcium concentrations, and tumor weight further support the concept that angiogenesis accompanies enhanced proliferative activity, endocrine hyperfunction, and increasing tumor burden. This relationship is biologically plausible, as rapidly proliferating tumors require continuous vascular expansion to ensure adequate oxygen and nutrient delivery, whereas newly formed blood vessels sustain the metabolic demands of continued tumor growth [10,11,12,13]. Conversely, increased proliferative activity may reflect expansion of hormonally active neoplastic cell populations capable of producing progressively greater amounts of PTH. Although these associations do not establish causality, they suggest that angiogenesis, tumor proliferation, endocrine hyperfunction, and tumor growth represent biologically interconnected processes rather than independent manifestations of malignancy.

Importantly, multivariable linear regression analysis demonstrated that preoperative serum PTH and calcium remained independent predictors of Ki-67 expression after adjustment for tumor size and tumor weight. From a clinical perspective, this observation emphasizes that biochemical markers may provide valuable information not only about endocrine function but also about the biological behavior of the tumor.

Previous studies have generally evaluated angiogenesis, proliferative activity, biochemical abnormalities, or adjunctive immunohistochemical parameter separately, whereas relatively few have explored the interrelationship among these parameters within the same cohort of parathyroid neoplasms [5,14,15,16,17,18,19,20,21]. Our findings therefore extend the current understanding of parathyroid carcinoma by demonstrating that angiogenesis, endocrine activity, tumor proliferation, and tumor burden are biologically interconnected rather than independent processes. Collectively, these observations support a model in which coordinated activation of neovascularization, cellular proliferation, endocrine hyperfunction, and progressive tumor growth contributes to the aggressive clinical phenotype of parathyroid carcinoma. Consequently, integration of biochemical, clinicopathological, and immunohistochemical parameters may provide a more comprehensive and biologically informed diagnostic framework than reliance on any individual marker alone, particularly in morphologically challenging parathyroid neoplasms.

### 4.5. Comparison with Established Diagnostic Biomarkers

Despite considerable advances in immunohistochemistry and molecular pathology, no single biomarker has demonstrated sufficient diagnostic accuracy to reliably distinguish parathyroid carcinoma from adenoma in all cases [5,17,18,19,20,21,22]. Loss of parafibromin expression remains one of the most reliable diagnostic indicators, particularly in tumors harboring CDC73 alterations, whereas Ki-67, galectin-3, PGP9.5, APC, and several additional markers have shown variable diagnostic performance across different studies [5,17,18,19,20,21,22]. In the present study, Ki-67 demonstrated a higher AUC than CD105-defined MVD, whereas CD105-defined MVD demonstrated strong discriminatory performance, particularly because of its 100% specificity at the study-specific ROC-derived cutoff. However, this finding should be interpreted cautiously given the small number of parathyroid carcinoma cases. These findings suggest that CD105-defined MVD and Ki-67 may be regarded as complementary adjunctive immunohistochemical parameters rather than standalone diagnostic markers, as they reflect distinct biological processes, namely tumor angiogenesis and cellular proliferation, respectively. Consequently, CD105-defined MVD should not be regarded as a replacement for established immunohistochemical markers but rather as an adjunctive biomarker that complements conventional histopathological evaluation. Integration of angiogenic, proliferative, and established diagnostic markers may therefore improve diagnostic confidence, particularly in morphologically challenging lesions, including atypical parathyroid tumors and carcinomas lacking unequivocal invasive features, in which reliance on a single biomarker may be insufficient [5,17,18,19,20,21,22]. This concept is consistent with current recommendations emphasizing integrated interpretation of histopathological, immunohistochemical, molecular, and clinicopathological findings when establishing the diagnosis of parathyroid carcinoma [5,17,18,19,20,21,22].

### 4.6. Strengths and Limitations

The present study has several strengths that enhance the validity and clinical relevance of its findings. To our knowledge, this study represents one of the larger single-center series evaluating CD105-defined microvessel density in parathyroid carcinoma and one of the few to investigate the relationship between tumor angiogenesis, proliferative activity, biochemical disease severity, and tumor burden within the same cohort of parathyroid neoplasms. Quantitative assessment of CD105-defined microvessel density using the widely accepted hotspot counting method originally described by Weidner, combined with ROC curve analysis, correlation analyses, and multivariable regression modeling, provided a comprehensive evaluation of the diagnostic and biological significance of tumor angiogenesis [12,13]. Furthermore, direct comparison with Ki-67 allowed us to explore the complementary roles of angiogenesis and cellular proliferation as reflected by adjunctive immunohistochemical parameters. The use of a homogeneous cohort from a single tertiary referral center ensured uniform tissue processing, immunohistochemical staining, and pathological evaluation throughout the study.

Nevertheless, several limitations should be acknowledged. First, the retrospective single-center design may limit the generalizability of the findings. The small number of parathyroid carcinoma cases also limits the reliability and generalizability of the ROC-derived thresholds; therefore, the reported cutoff values should be considered exploratory and require validation in larger, independent cohorts. An additional limitation relates to the use of archival FFPE tissue. Although the specimens were stored under routine institutional archival conditions and preservation of CD105 immunoreactivity was assessed using internal endothelial structures as positive controls, prolonged storage may still result in variable antigen preservation and potential epitope degradation. This pre-analytical variability may have influenced immunohistochemical staining intensity and, consequently, MVD measurements. Nevertheless, the use of standardized immunohistochemical procedures, together with internal endothelial controls and external positive and negative controls, was intended to minimize the potential impact of this variability. Second, although microvessel density was assessed using the standardized hotspot counting method described by Weidner, manual vessel counting remains susceptible to observer-dependent variability despite the use of predefined evaluation criteria. Finally, additional angiogenic markers, such as VEGF, CD31, and CD34, and molecular alterations potentially relevant to parathyroid carcinoma, including CDC73 alterations, were not evaluated, precluding direct comparison of CD105-defined MVD with other angiogenic and molecular parameters [5,17,18,19,20,21,22].

Future multicenter studies involving larger international cohorts are warranted to validate the diagnostic performance of CD105-defined microvessel density, establish standardized cut-off values applicable across different institutions, and confirm the reproducibility of these findings. Integration of additional angiogenic markers, molecular characterization, long-term clinical follow-up, and emerging digital pathology and artificial intelligence-assisted image analysis may further improve the objectivity and standardization of microvessel density assessment while providing a more comprehensive understanding of the diagnostic, biological, and potential prognostic significance of tumor angiogenesis in parathyroid carcinoma [28,29,30].

## 5. Conclusions

The present study demonstrates that tumor angiogenesis represents an important biological feature of parathyroid carcinoma and is significantly increased compared with parathyroid adenoma. Quantitative assessment of CD105-defined microvessel density showed strong discriminatory performance and was closely associated with proliferative activity, biochemical disease severity, and tumor burden, supporting the concept that angiogenesis is an integral component of the aggressive biological phenotype of parathyroid carcinoma.

CD105-defined MVD is a potentially useful adjunctive parameter reflecting tumor angiogenesis that may contribute to the differential diagnosis of parathyroid carcinoma and parathyroid adenoma. It should not replace established histopathological criteria or validated immunohistochemical biomarkers but may provide supportive information within a multiparametric diagnostic approach, particularly in morphologically challenging cases. Integration of quantitative angiogenic assessment with conventional histomorphology, clinicopathological findings, and established biomarkers may improve diagnostic confidence; however, the study-specific ROC-derived cutoff should be considered exploratory and requires validation in larger, independent cohorts.

Further prospective multicenter studies involving larger patient cohorts, long-term clinical follow-up, and integration of molecular biomarkers are warranted to validate these findings and to further define the diagnostic and potential prognostic significance of tumor angiogenesis in parathyroid carcinoma.

## Figures and Tables

**Table 1:** Demographic characteristics of the study population. (A) Distribution of study participants according to sex. (B) Age of study participants at the time of surgery.

**(A)**						
**Diagnosis**		**Sex**			**Total**	
		**Male**		**Female**		
Carcinoma	N	6		4	10	
	%	60.0		40.0	100.0	
Adenoma	N	10		30	40	
	%	25.0		75.0	100.0	
Control group	N	1		9	10	
	%	10.0		90.0	100.0	
Total	N	17		43	60	
	%	28.3		71.7	100.0	
(**B**)						
**Diagnosis**	**N**	**Mean**	**SD**	**Median**	**Minimum**	**Maximum**
Carcinoma	10	53.00	14.17	53.50	33.00	76.00
Adenoma	40	54.30	12.28	55.50	15.00	77.00
Control group	10	45.70	12.88	43.50	23.00	64.00
Total	60	52.65	12.87	53.50	15.00	77.00

**Table 2:** Ki-67 values according to histopathological diagnosis.

Diagnosis	n	Mean	SD	Median	Minimum	Maximum
PC	10	1024.27	564.19	785.15	523.43	2069.95
PA	40	334.66	161.71	297.41	130.86	713.77
Total	50	472.58	396.13	374.73	130.86	2069.95

**Table 3:** CD105-defined microvessel density according to histopathological diagnosis.

Diagnosis	n	Mean	SD	Median	Minimum	Maximum
PC	10	830.36	224.06	901.14	410.42	1017.13
PA	40	447.22	211.63	431.24	0.00	773.26
Total	50	523.85	262.36	499.64	0.00	1017.13

**Table 5:** Selected Pearson correlations between angiogenesis, proliferative activity, biochemical variables, and tumor morphology.

Variable Pair	Pearson r	*p* Value	Interpretation
MVD vs. Ki-67	0.389	0.005	Moderate positive association
MVD vs. preoperative PTH	0.534	<0.001	Moderate positive association
MVD vs. preoperative calcium	0.406	0.003	Moderate positive association
MVD vs. tumor diameter	0.277	0.052	Borderline, not statistically significant
MVD vs. tumor weight	0.356	0.011	Positive association
MVD vs. preoperative phosphate	−0.070	0.627	No significant association
MVD vs. age	−0.001	0.997	No significant association
Ki-67 vs. preoperative PTH	0.562	<0.001	Strongest Ki-67 association
Ki-67 vs. preoperative calcium	0.571	<0.001	Strongest Ki-67 association
Ki-67 vs. tumor diameter	0.378	0.007	Positive association
Ki-67 vs. tumor weight	0.382	0.006	Positive association

## Data Availability

Data supporting the findings of this study are available from the corresponding author on reasonable request. The dataset is not publicly available due to patient privacy and ethical restrictions.

## Abbreviations

The following abbreviations are used in this manuscript:

PC	Parathyroid carcinoma
PA	Parathyroid adenoma
PHPT	Primary hyperparathyroidism
MVD	measuring microvessel density
ROC	Receiver operating characteristic
VEGF	vascular endothelial growth factor
FFPE	formalin-fixed, paraffin-embedded
MEN	multiple endocrine neoplasia
PTH	Parathyroid hormone
H&E	Hematoxylin and eosin
ABC	avidin–biotin complex
AUC	area under the curve
DAB	3,3′-diaminobenzidine
SD	standard deviation
IQR	Interquartile range
